# A process evaluation of a randomized-controlled trial of community gardening to improve health behaviors and reduce stress and anxiety

**DOI:** 10.1038/s41598-024-63889-w

**Published:** 2024-06-13

**Authors:** Eva Coringrato, Katherine Alaimo, Jenn A. Leiferman, Angel Villalobos, Hannah Buchenau, Erin Decker, Lara Fahnestock, Pallas Quist, Jill S. Litt

**Affiliations:** 1 Department of Environmental Studies, University of Colorado Boulder, 4001 Discovery Drive, Boulder, CO 80303 USA; 2 Department of Food Science and Human Nutrition, G. Malcolm Trout Building, Room 208C, Michigan State University, 469 Wilson Road, East Lansing, MI 48824 USA; 3 Department of Community and Behavioural Health, Colorado School of Public Health, Mail Stop B-119, Room W 3140, 13001 East 17th Place, Aurora, CO 80045 USA; 4 Denver Urban Gardens, 1031 33rd Street, Suite 100, Denver, CO 80205 USA

**Keywords:** Cancer prevention, Psychology and behaviour, Environmental social sciences, Risk factors, Cancer, Cardiovascular diseases, Anxiety

## Abstract

As part of the Community Activation for Prevention (CAPS) randomized controlled trial (RCT) of community gardening, we conducted a process evaluation to assess the implementation of a community gardening intervention over nine months, as measured by reach, fidelity (delivery, receipt, enactment), and acceptability. Evaluation instruments included repeated semi-structured interviews with study participants, direct observation of community garden sites, and an exit survey of participants. Primary outcomes were diet, physical activity, and anthropometry; secondary outcomes were stress and anxiety. The CAPS trial included 291 participants (19% non-white; 34% Hispanic/Latino; 35% without a college degree; 58% with income < $50,000 per year). Intervention delivery and receipt were high for environmental supports. Garden social events were offered by 73% of gardens, although only 48% of intervention participants reported attending these events. Of the 145 participants assigned to the gardening intervention, 97 (67%) reported gardening the entire season and reported visiting the community garden a median of 90 min per week (range: 0–840). Of the participants who completed the exit survey (48%), 89% were highly satisfied with the overall garden experience. The CAPS trial was favorably received and implemented with high fidelity, supporting the validity of the trial outcomes. These findings suggest that community gardens are a viable health promotion strategy that can be successfully implemented among new gardeners from diverse backgrounds. Strategies that engage new gardeners in the social aspects of the garden environment and connect gardeners with garden “mentors” or “buddies” to ensure new gardeners achieve success in their first years of gardening are recommended.

Trial registration: NCT03089177. Registered 24 March 2017, https://clinicaltrials.gov/study/NCT03089177.

## Introduction

Community and allotment gardens have been widely adopted across the world, and the literature is rich with research on the benefits of gardens on health and well-being^1^. These studies have shown that community gardens offer a promising system through which we can learn about lifestyle interventions to promote health behavior change, psychosocial and physical health outcomes across diverse populations in different social, cultural, geographic, economic, and environmental contexts. Moreover, gardens have been shown to influence health and wellbeing by activating emotional (intrapersonal), social (interpersonal), and environmental processes that are central to healthy lifestyle habits^2,3^. Eating well and maintaining regular physical activity, for example, help prevent cancer, heart disease, diabetes, and other chronic diseases^4^.

Past observational, quasi-experimental, and qualitative studies have shown that vegetable gardening was feasible in the home^5,6^, community^7^, and hospital^8^ contexts. Participants in previous gardening studies reported high levels of satisfaction with these interventions, indicating that gardening was generally acceptable as a behavioral intervention^5,6,8^. However, the designs of these studies preclude our ability to determine whether the positive effects of gardening were the result of the garden itself or whether people seeking improvements to their health and wellbeing self-selected into the garden because they already have these healthy habits. Thus, there is a need for experimental studies of gardens with randomization to improve our causal understanding of how gardens can improve health and well-being^1,9^. Moreover, co-designing a process evaluation in tandem with a randomized controlled trial can serve to contextualize and validate the trial outcomes and guide implementation^10,11^.

The Community Activation for Prevention (CAPS) randomized controlled trial assessed whether participation in community gardens improved primary outcomes of diet, physical activity, and anthropometry and secondary outcomes of perceived stress and anxiety among a multi-ethnic, mixed-income population of adults^12–14^. The study found significant time-by-treatment effects for fiber intake (p = 0.034), moderate-to-vigorous physical activity (p = 0.012). No significant time-by-treatment effects were found for combined fruit and vegetable intake, Healthy Eating Index, sedentary time, body mass index, and waist circumference. Difference score models showed greater reductions in perceived stress and anxiety among participants in the intervention group than among those in the control group^13^. The process evaluation aimed to understand the CAPS gardening intervention implementation by examining the reach, fidelity, and acceptability of the intervention within the context of a randomized controlled trial and specifically how new gardeners experienced the intervention.

## Methods

### CAPS trial

The CAPS trial collected data from 2017 through 2020 and examined whether community garden participation improved diet, physical activity, and body mass index, and reduced perceived stress and anxiety. The trial protocol and outcomes are reported elsewhere^12,13^. The theoretical basis of this study was informed by multiple theoretical frameworks including self-determination theory^15^, social cognitive^16^, and ecological system^17,18^ theories and results from past empirical studies among Denver gardeners^3,19–22^.

The RCT was conducted within a critical realist framework to capture the social and cultural context of community gardening. A concurrent process evaluation was designed in tandem with the trial to understand intervention reach, fidelity, and acceptability and allow us to interpret findings and understand their application more broadly^23^. The trial occurred over three waves, each lasting approximately one year, and included a complete gardening season for each study participant. Criteria for inclusion were: 1) not having gardened in the last two years, 2) being 18 years of age or older, and 3) the ability to give consent in English or Spanish. Participant outcome data were collected at baseline before the gardening season and before random allocation (T1), during fall harvest (T2), and during the winter post-intervention (T3).

### Recruitment

For this trial, garden waiting lists were used as the basis for the recruitment. The wait lists pre-existed at each of the gardens. Study staff, in cooperation with DUG staff, invited individuals on the garden waiting lists to join the study. This recruitment process involved 37 community gardens spanning the greater Denver metropolitan area. Details about recruitment are reported elsewhere^12–14^.

### Informed consent and ethics approval

All study participants provided written informed consent before enrolment and randomisation. The study protocol and informed consent were approved by the University of Colorado Boulder Institutional Review Board (Protocol # 16-0644). The trial was registered on March 24, 2017 at ClinicalTrials.gov (NCT03089177). All methods were performed in accordance with the Standards for Reporting Implementation Studies (StaRI)^24^, the CONSORT guidelines^13^ and in accordance with the Declaration of Helsinki.

### Trial setting

The trial was conducted in partnership with Denver Urban Gardens (DUG), a non-profit organization that builds and manages 200 community gardens in the Denver, Colorado metropolitan area. We worked with community garden leaders to secure two to six plots per garden for study participants. For each plot secured, the study paid $50 to garden-specific spending accounts ($100-$300 per garden). Participants randomized to a garden plot were provided the tools, resources, and education typically offered to community gardeners in the DUG network, including a garden plot, seeds, plant seedlings, an introductory gardening course, and opportunities for social interaction, mentorship, and community-building specific to each garden site, as described in Table 1. The “Grow a Garden” class provided basics on growing food and caring for the garden plots. The class was offered to all participants in the intervention group. Additionally, participants received planting guides and worksheets for growing produce in dry climates. All aspects of the intervention mirrored current practices by DUG. Study participants were treated similarly to community gardeners in the DUG network.

**Table 1:** Core elements of the CAPS intervention included provision of garden plots, seeds and plant seedlings, introductory gardening classes, and social events, community building, and mentorship at each garden site.

Core elements of the CAPS intervention^a^	Description
Garden plots	A standard community garden plot was provided to all intervention participants with plot fees paid by the study
Seeds and plant seedlings	Intervention participants were provided seeds and seedlings to grow in the garden plots. All intervention participants were offered 10 seedlings and a bag of seeds containing at least 10 seed packets; Seedlings included tomatoes, peppers, broccoli, cabbage, cauliflower, eggplant, and basil. Seed packets were variable but typically contained: bush beans, carrots, lettuce varieties, kale, spinach, zucchini, radish, collard greens, scallions, squash, Swiss chard, and cilantro
Introductory gardening class	An introductory gardening classes taught by a Master Community Gardener through Denver Urban Gardens and conducted in both English and Spanish were offered to all intervention participants
Social events, community building, and mentorship	Opportunities for social interaction, community building, and mentorship were offered via garden social events and other formal and informal opportunities for social interaction within each garden site

^a^Control participants were offered the same core elements after they completed participation in the trial.

### Data collection

Process evaluation data were collected via semi-structured interviews with participants, direct observation of community garden sites, and surveying of participants to assess reach, fidelity, and acceptability. Garden leaders provided information about garden sites including the number and size of plots. Data collection occurred concurrently with intervention implementation. All participants were contacted for two process evaluation interviews. The first interview occurred at week 15 of the intervention period (August) to capture peak gardening season and the second interview occurred at week 25 (November) to reflect the remainder of the gardening season. These interviews served both to assess elements of intervention fidelity and to improve participant retention. Intervention participants were asked about their experiences, habits, and routines in the garden including preparing the garden plot, types of fruits and vegetables planted and harvested, general gardening routine, frequency and intensity of gardening, challenges to participating in the garden, communication and interactions with garden leadership, interactions with other gardeners, and educational event attendance. Control participants were contacted concurrently for a check-in to maintain parity in attention and were asked about their general activities since the most recent data collection time point. The interview instrument is available in Supplementary Information. Bi-annual garden audits, conducted in July and October, captured the physical appearance and upkeep of gardens. Finally, an exit survey was implemented via email at the conclusion of the study for all participants to assess satisfaction with various components of the community garden experience and study participation.

### Measures

We assessed intervention reach, fidelity, and acceptability as detailed a priori in the trial protocol (see Table 2)^12^. Reach is defined as the proportion of the target population that participated in the intervention^25^. Measurements included the number and demographics of screened and eligible participants and the number and location of garden sites. Neighborhood level sociodemographic characteristics surrounding garden sites were assessed by spatial analysis of community garden sites. Sites were mapped by the poverty level of the census tracts at their location^26,27^.

**Table 2:** Measurement tools, items, and timeline for each process evaluation element: reach, fidelity, and acceptability.

Construct	Measurement tool(s)	Measurement items	Timeline				
			Screening	Baseline (T1) health visit	Mid-season	Post-season	Post-intervention
Reach^a^							
	Recruitment questionnaire	Participant demographics	✓				
	Garden recruitment survey	Community garden location	✓				
Fidelity^b^							
Delivery and receipt	Garden registration survey	Number and size of garden plots provided	✓				
	Garden audits	Quality of garden plots			✓	✓	
		Availability of garden resources			✓	✓	
	Participant interviews	Quality of garden plots			✓	✓	
	Seeds and seedling order forms	Seeds and seedlings provided	✓				
	Calendar of events	Introductory gardening class provided and attended	✓				
	Participant interviews	Communication from garden leadership			✓	✓	
		Garden events provided			✓	✓	
Enactment	Participant interviews	Gardening frequency and duration			✓	✓	
		Gardening routine			✓	✓	
		Degree of planting and harvesting			✓	✓	
		Barriers and challenges to participation			✓	✓	
		Interaction with other gardeners			✓	✓	
		Garden events attended			✓	✓	
Acceptability^c^							
	Recruitment questionnaire	Participant discontinuation	✓	✓	✓	✓	
	Exit survey	Overall satisfaction with the community garden					✓
		Satisfaction with garden plot					✓
		Satisfaction with seeds and seedlings					✓
		Satisfaction with gardening class					✓
		Satisfaction with opportunities to interact with other gardeners					✓
		Satisfaction with garden leadership					✓
		Satisfaction with garden resources					✓
		Satisfaction with respect for plants and property					✓
		Satisfaction with garden events					✓
		Satisfaction with convenience of the garden					✓
		Satisfaction with opportunities to learn from other gardeners					✓

^a^As defined in Saunders et al.^24^, ^b^as defined in Bellg et al.^28^ and Moore et al.^21^, ^c^as defined in Bowen et al.^31^ and Sekhon et al.^30^.

Fidelity assesses whether an intervention is implemented as intended^10,28^ and commonly examines delivery, receipt, and enactment of intervention components^29,30^. Delivery assesses whether core intervention components are uniformly administered by the study team according to the study protocol. Receipt assesses the level of uptake of intervention components and resources by participants. Enactment assesses the extent to which participants engaged with core components of the intervention. Elements of CAPS delivery and receipt included provision and quality of garden plots, garden resources, seeds, plant seedlings, gardening classes, and opportunities for social interaction within the garden, including garden events. Elements of enactment included gardening frequency, duration, and routine, planting and harvesting, barriers and challenges to participation, interaction with other gardeners, and event attendance. Gardening frequency was computed as the average number of times a participant visited the garden per week, and intensity of gardening was computed as the average time spent gardening during each visit.

Acceptability assesses the extent to which participants consider the intervention to be appropriate^31^, including participant satisfaction with intervention components^32^. Participant satisfaction was assessed via an exit survey and study dropout was monitored throughout the trial. Participants rated their satisfaction of the garden plot, seeds, seedlings, gardening classes, opportunities for social interaction within the garden, garden leadership, garden resources, events, convenience getting to the garden, and opportunities to learn from other gardeners.

### Data analysis

We employed a mixed-methods approach that utilized qualitative and quantitative data. Participant interviews included structured and semi-structured questions. Interviews were audio recorded and then responses to semi-structured questions were summarized by research staff. For a subset of interviews that were not audio-recorded, interviewers recorded responses by hand. Interview transcripts and summaries were first cleaned for clarity and completeness. Responses to semi-structured questions were analyzed for thematic content by the first author. Themes were iteratively identified for each question and coded summaries were then mapped to process evaluation components. Summaries of the number of participants addressing a particular evaluation component were tallied. For quantitative variables, summary statistics including means, medians, standard deviations, and percentages were computed using STATA 13^33^. A two-tailed Fisher’s exact test with a significance level of 0.05 was used to evaluate statistical differences between categorical variables.

## Results

### Reach

#### Participants

The CAPS trial consented 291 participants. There were no significant differences in demographic characteristics between individuals who completed screening for the trial and consented to participate and individuals who completed screening and declined participation in the trial (see Table 3). The majority (82%) of study participants were female. Nineteen percent of participants identified as non-white, 34% of participants identified as Hispanic or Latino, 13% of participants spoke Spanish as their primary language, 58% had an annual household income below $50,000, and 35% of participants did not have a college degree. The racial, ethnic, and income demographics of CAPS participants largely reflected those of the Denver Metropolitan Area, where 19% of residents identified as non-white, 29% of residents identified as Hispanic or Latino, and the median annual household income was $68,592 during the time of trial^34^.

**Table 3:** Demographic characteristics of individuals who completed screening for the trial and consented to participate (n = 291) and individuals who completed trial screening and declined participation (n = 177).

Demographic characteristics of screened individuals		
	Participants (N = 291)	Nonparticipants (N = 177)
Age, mean (range)	41 (20–77)	40 (19–76)
Gender		
Female (%)	238 (82%)	118 (67%)
Male (%)	53 (18%)	31 (18%)
Do not identify as male or female (%)	1 (0%)	0
Missing (%)	0	28 (16%)
Race		
White (%)	236 (81%)	113 (64%)
Black/African American (%)	18 (6%)	14 (8%)
Asian (%)	12 (4%)	3 (2%)
Native Hawaiian or other Pacific Islander (%)	0	0
American Indian or Alaska Native (%)	7 (2%)	5 (3%)
Other (%)	15 (5%)	13 (7%)
Refused (%)	1 (0%)	0
Do not know (%)	2 (1%)	0
Missing (%)	0	29 (16%)
Ethnicity		
Hispanic/Latino (%)	99 (34%)	59 (33%)
Not Hispanic/Latino (%)	190 (65%)	86 (49%)
Refused (%)	2 (1%)	0
Missing (%)	0	32 (18%)
Preferred language		
English (%)	253 (87%)	138 (78%)
Spanish (%)	38 (13%)	34 (21%)
Missing (%)	0	5 (3%)

### Community garden sites

Thirty-seven community gardens, situated in four counties in the Denver Metropolitan Area, participated in the CAPS trial over three waves. Forty-four percent of participating community gardens were in census tracts with more than 20% of the population living below the federal poverty level^34^.

### Fidelity

Fidelity assessed intervention delivery, receipt, and enactment. Results are described below and in Table 4. Unless otherwise indicated, percentages are reported for intervention participants (n = 145).

**Table 4:** Summary of elements of fidelity of delivery, receipt, and enactment among intervention participants (N = 145).

Fidelity elements	n (%)^a^
Delivery and receipt	
Plots assigned	
Yes	145 (100%)
No	0 (0%)
Seeds and seedlings delivered	
Yes	145 (100%)
No	0 (0%0
Classes offered	
Yes	145 (100%)
No	0 (0%)
Attended gardening class	
Yes	65 (45%)
No	40 (28%)
Missing/refused	40 (28%)
Gardening class was helpful	
Yes	46 (32%)
No	9 (6%)
Did not attend a gardening class	40 (28%)
Missing/refused	50 (34%)
Adequate access to water	
Yes	81 (56%)
No	14 (10%)
Missing/refused	50 (34%)
Concerns about soil quality	
Yes	21 (14%)
No	63 (43%)
Missing/refused	61 (42%)
Felt safe in the garden	
Yes	83 (57%)
No	6 (4%)
Missing/refused	56 (39%)
Getting started was difficult	
Yes	65 (45%)
No	35 (24%)
Missing/refused	45 (31%)
Events were held at the garden	
Yes	75 (52%)
No	28 (19%)
Missing/refused	42 (29%)

Enactment	
Planted by August	
Yes	105 (72%)
No	0 (0%)
Missing/refused	40 (28%)
Harvested by August	
Yes	78 (54%)
No	26 (18%)
Missing/refused	41 (28%)
Garden events attended	
All events	8 (6%)
The majority of events	12 (8%)
Some of the events	28 (19%)
None of the events	53 (37%)
Missing/refused	44 (30%)
Frequency of interaction with other gardeners	
Daily	39 (27%)
Weekly	39 (27%)
Monthly	5 (3%)
Once in a while	19 (13%)
Never	1 (1%)
Missing/refused	42 (29%)
Frequency of interaction with garden leadership	
Daily	1 (1%)
Weekly	37 (26%)
Monthly	28 (19%)
Once in a while	22 (15%)
Never	2 (1%
Missing/refused	55 (38%)

^a^ N=145, corresponding to the total number of intervention participants across all study waves.

### Delivery and receipt

#### 1. Garden plots

Intervention participants were provided a single, in-ground garden plot in a participating community garden near their home. In participating gardens, the average square footage of a garden plot was 116 square feet (range: 32 to 200 square feet) and community gardens, on average, contained 31 plots (range: 12–100). Garden audits revealed that an average of 20% of plots across all garden sites were empty or abandoned, while the majority (66%) appeared to be well-kept based on the health of plants (dead plants, presence of pests, etc.) and the presence of weeds.

The majority (n = 81, 56%) of participants reported having adequate access to water in the garden (Table 4). Less than one quarter of participants (n = 21, 14%) reported issues with soil quality in their plots (Table 4). Participants cited issues including presence of trash (n = 8, 6%), nutrient deficiencies (n = 7, 5%), presence of clay, sand, or rocks (n = 7, 5%) and contamination with pesticides or mold (n = 3, 2%). The majority of participants (n = 83, 57%) reported feeling safe at the garden (Table 4). Those that reported safety issues cited concerns about personal safety (n = 8, 6%), lack of lighting at the garden (n = 8, 6%), theft of garden produce (n = 4, 3%), and vandalism (n = 2, 1%).

Nearly half of the participants (n = 65, 45%) described getting started in the garden as difficult (Table 4) and reported feeling overwhelmed upon first receiving their garden plot, particularly if the plot was overgrown with weeds. Some participants (n = 20, 14%) felt that they did not have the knowledge necessary to prepare their plot for the gardening season. A few participants (n = 9, 6%) reported spending more time than expected preparing their plots for the growing season, and some noted that the work was physically taxing (n = 14, 10%). Some participants (n = 19, 13%) reported that being confronted with a large plot full of weeds on their first day in the garden was intimidating. Participants who received in-garden support from leaders or other gardeners reported feeling less intimidated when beginning to garden. One participant described her experience working on the plot for the first time:

“The plot was full of weeds, and it was a lot of work to get the soil ready. I felt overwhelmed by the size of the plot.” (Participant 79)

All intervention participants were connected to garden leaders at their assigned garden via email or phone at the beginning of the gardening season. Occasionally, participants reached out to study staff for help in their garden, particularly at the beginning of the season. To follow standard practices established by DUG, staff encouraged and assisted participants in connecting with their garden leader or Master Community Gardeners (MCG) available through DUG. In instances where participants did not have sufficient support and considered dropping out, study staff met participants at garden sites to help, using standard practices established by DUG staff and MCGs.

Additionally, some participants were assigned to garden plots later than others due to recruitment delays, T1 data collection requirements, and garden opening delays due to weather or construction. Some of these participants (n = 15, 15%) mentioned that due to these delays, they could not attend the garden orientation, limiting connections with other gardeners, shortening the gardening season, and impacting the overall garden productivity. One participant noted:

“Getting started late was a challenge-the plants didn't have time to grow and are comparatively smaller than those belonging to other gardeners. The zucchini is growing but very slowly and too small to pick.” (Participant 412)

#### 2. Seeds and seedling

Seeds and seedlings were provided to all intervention participants at DUG’s central office during gardening classes and other specified days (see Table 1). Participants could purchase additional seeds and seedlings if desired, and many gardens provided seeds to gardeners at no cost. Alternative arrangements were made for participants who were not able to travel to the DUG office.

#### 3. Introductory gardening classes

Introductory gardening classes were conducted in-person in either English or Spanish on weekdays, evenings, and weekends to accommodate participants’ schedules. Control participants were offered classes the following year. Attendance fees were covered by the study ($40 at the time of trial). Classes, approximately three hours in duration, included a lecture, print materials, and the opportunity to ask questions of MCGs.

Among intervention participants, 45% reported attending introductory gardening classes at the beginning of the gardening season (Table 4). Of those who attended, 71% (n = 46) found the classes helpful for getting started in their gardens (Table 4). Several participants, however, felt the classes did not prepare them to garden, leading to feelings of insufficient gardening skills and knowledge (n = 6, 4%), as described below:

“They [class instructors] did not go over what tools I should use or how I should get started in my plot. They told me what plants I should start planting and when, but they did not give me any specifics on how to weed or turn my soil and add compost. I was very confused about that.” (Participant 283)

#### 4. Communication and social opportunities at the garden

##### (a) Communication from garden leadership

Garden leaders, as volunteers, are provided leadership training and support from DUG, in addition to an annual garden leader symposium to foster an exchange among garden leaders and build leadership skills. Garden leaders were an important bridge between DUG and study participants. Once study participants were assigned to gardens, they received garden-specific email and text messages and were invited to social events, including garden workdays.

Participants were asked about the type and frequency of communication between leaders and gardeners. Gardeners reported adequate communication with garden leaders, with many participants reporting weekly or monthly communication (n = 65, 45%) and a smaller fraction reporting communication “once in a while” (n = 22, 15%). Only two participants (1%) reported never having communication with garden leaders. Some Spanish-speaking gardeners noted communication challenges with garden leaders who did not speak Spanish. Communication ranged from routine communication with gardeners to ad-hoc to address issues in the garden or to announce events.

Most participants characterized garden leadership positively. Participants commonly described garden leaders as “sweet,” “friendly,” and “encouraging.” Nearly one-quarter of participants (n = 33, 23%) recalled receiving a garden orientation from the garden leader and found garden leaders to be “helpful” (n = 29, 20%). Responses to semi-structured questions revealed that a subset of participants (n = 18, 12%) described their garden leader as largely “absent” from the garden, noting that they rarely or never saw garden leaders at the garden, or received communication from leaders only at the beginning of the season. While some of these participants appeared dissatisfied with the lack of interaction with garden leadership, other participants found community and gardening resources through other avenues. When asked about interacting with garden leaders, one participant described:

“They [garden leaders] showed us around and had us sign all the paperwork, pointed out the plots that were available, and asked if we had any questions. They told us the rules and regulations. We were on our own after that. There were initially some days or times that the garden leaders were going to be there, but I haven't been able to go to those. They are very friendly, helpful, and accessible. I have been able to ask them a question. I do my own research as well and look things up.” (Participant 264)

##### (b) Events offered at the garden

While not required, many gardens hosted events such as orientations, workdays, gardening workshops, or potlucks. Just over half of participants (52%) confirmed that events were held at their garden. Participants who reported that their garden did not host events represented 16 community gardens (43% of community gardens included in the study).

### Enactment

#### 1. Engagement with the community garden plot

##### (a) Frequency and intensity of gardening

The overwhelming majority of intervention participants (n = 116, 80%) reported doing some community gardening during their participation in CAPS. Of those 116 participants, 97 participants (84%) reported gardening for a full gardening season (May through October). Overall, the median time spent gardening was 90 min (range 0–840 min) per week during the gardening season. Most participants (78%) visited the garden at least twice per week and over half of participants (60%) reported spending at least 30 min at the garden per visit.

##### (b) Gardening routine

When asked about their gardening routine, nearly all participants described weeding and watering as part of their regular activities in the garden. Several participants (n = 24, 17%) described harvesting from their plots as a part of their routine. Some participants (n = 22, 15%) noted that they regularly cared for communal spaces in the garden in addition to their own plot. A few participants (n = 11, 8%) described socializing with other gardeners as a part of their routine in the garden and some (n = 9, 6%) mentioned helping other gardeners with their plots. A few participants (n = 11, 8%) noted spending leisure time in the garden to enjoy the garden environment.

##### (b) Planting and harvesting

Intervention participants reported both planting and harvesting from their garden plots. When interviewed in August each wave, 105 (72%) of all intervention participants had planted in their garden and more than half of the participants (54%) had harvested produce from their garden plots (Table 4). Overall, of those who gardened, 97% of intervention participants planted vegetables, 24% planted herbs, and 24% planted fruit. By August, 54% of participants reported harvesting from their garden plot, including vegetables (69%), herbs (16%), and fruit (13%). Commonly planted and harvested garden produce included: squash, peppers, tomatoes, cruciferous vegetables, beans, peas, and leafy greens.

##### (c) Barriers and challenges to participating in the community garden

Participants were asked if they experienced any challenges getting to or participating in the community garden during the trial. Participants reported challenges including time or conflicting priorities (n = 33, 23%), distance to the garden (n = 17, 12%), traveling or vacationing (n = 8, 6%), childcare/supervision (n = 6, 4%), and weather (n = 6, 4%). Participants who struggled to find time to garden often mentioned that busy work schedules, shift work, working multiple jobs, or caring for family members took up much of their time. While study staff aimed to place participants in gardens near their homes, nonetheless, some participants experienced challenges getting to the garden. Several participants reported driving to their garden and mentioned that this posed an additional barrier to the frequency of their garden visits.

#### 2. Social opportunities at the garden

##### (a) Interactions with other gardeners

Most participants (n = 78, 54%) reported having daily or weekly interactions with other community gardeners (Table 4). When asked to describe their interactions with other gardeners, most participants (n = 72, 50%) mentioned engaging in small talk with other gardeners, and many participants (n = 47, 32%) described other gardeners as friendly. Several participants (n = 38, 26%) mentioned receiving help and advice from other gardeners. Some participants (n = 18, 12%) also noted that they exchanged produce with other gardeners. One gardener described interacting with other gardeners:

“We [the participant and other gardeners] loved sharing stories, sharing tips, and sharing produce. We would mostly talk about gardening because that was what we all had in common.” (Participant 264)

A subset of participants (n = 19, 13%) described a lack of social interaction with other gardeners, and some participants (n = 5, 3%) noted that there seemed to be little participation in their garden overall, and consequently, it was rare to encounter or interact with other community gardeners. Other participants in more active gardens noted feeling shy around other gardeners, not wanting to disturb others while gardening, feeling excluded from or intimidated by the established gardening community, or feeling like a newcomer. One participant described having little interaction with other gardeners:

“It was very rare for me to talk to any of the gardeners. I didn't feel like they were approachable. I didn't feel comfortable interacting with them, maybe it was a problem with me- I am not sure. Since I came in so late in the season, I wasn't acquainted with anyone.” (Participant 515)

##### (b) Event attendance

One third of participants (n = 48, 33%) reported attending garden events (Table 4). Most of these participants who attended garden events reported attending workdays (n = 21, 44%), with a smaller number of reporting attending potlucks (n = 5, 10%), classes held at their garden (n = 5, 10%), or multiple types of events (n = 6, 13%). Participants who did not attend events reported that the time of the events did not align with their schedule or that they did not hear about the events in time to attend.

### Acceptability

#### Participant discontinuation

A total of 63 participants (22%) dropped out of the trial after randomization, well within our planned loss to follow up of 30%^12^, 36 participants from the intervention group (25%) and 27 participants from the control group (18%). Causes of participant dropout included: did not accept randomization, chose to not continue, moved away, participation was too challenging, and reasons unknown^13^.

#### Participant satisfaction with the intervention

Almost half of the intervention participants (48%) responded to exit survey questions about the acceptability of the intervention and their satisfaction with different components of the community gardening experience. Intervention participants who responded reported high overall satisfaction with the garden (89%) and high levels of satisfaction with the core components of the intervention, namely quality of the garden plot (84%), seeds and seedlings provided by the study (97%), gardening classes (76%), and opportunities to interact with other gardeners (73%) and with garden leadership (70%). Of those who responded, 97% were satisfied with water availability, 94% were satisfied with the tools provided, 80% were satisfied with the lighting in their garden, 94% were satisfied with garden safety, and 86% were satisfied with the level of respect for plants and property in the garden. Respondents also reported moderate-to-high levels of satisfaction with other intervention components: 79% were satisfied with the convenience of getting to the garden, 60% were satisfied with events held at the garden, and 64% were satisfied with opportunities to learn from other gardeners. No significant differences in participant satisfaction were observed by demographic groups, including gender, age, race, ethnicity, education, and income.

## Discussion

The results of this process evaluation, and informed by previously published RCT results^13^, helped us understand the implementation and contextual factors of an urban community garden program to promote diet, physical activity, and emotional wellbeing. Using a well-planned evaluation framework that adopted a realist approach and was conceptualized as part of the initial planning and design of the intervention, we showed that the garden intervention achieved high implementation levels for intervention reach, delivery, and receipt^29^. We considered a range of factors that affected implementation, including training and support offered to intervention participants and communication and management elements, and how these factors interacted to influence the deployment of the intervention and participant experiences. For example, inside the project team, regular meetings improved internal communication and helped address challenges during trial implementation, created synergy among investigators and field staff and across the various intervention elements, and improved adherence to study protocols. CAPS participants showed high engagement with and acceptance of the intervention activities, lending validation to the overall assessment of implementation for this trial.

The process evaluation revealed that gardening was acceptable for people across different social and economic groups and that it was a viable activity for people who were new to gardening. Examining these factors was an important to overarching objectives of the study. That is, the combined mixed methods approach of the CAPS trial and process evaluation were designed through an equity lens, to understand how gardening interventions can reduce health inequalities linked to socio-economic status and ethnicity. The large sample of the trial allowed us to understand the causal pathway between garden participation and primary health and secondary psychosocial outcomes. The process evaluation enabled us to examine the barriers and enablers to gardening participation. The study came together with the input of multiple third sector organizations, including Denver Urban Gardens, and community participation in the design, implementation, and evaluation of the study. These diverse perspectives were critical in ensuring that the research findings could inform practice to improve health equity^35^. The results of this evaluation add to the growing body of literature showing that gardening is a feasible and acceptable health-promotion intervention among diverse populations^5–8^.

This evaluation captured the varied ways participants engaged with the community garden environment and illuminated where interventions and garden organizations could better support new gardeners in adopting gardening. To promote gardening as a health intervention for people new to gardening from different social, economic, and demographic groups, a mosaic of support is needed. This approach includes intrapersonal (e.g., competency to garden—that is, the skills and self-efficacy needed to “get started” and “keep up with it”), interpersonal (e.g., shared learning and relatedness), and environmental supports (e.g., structural resources and physical accessibility)^2,36^.

While the core gardening elements worked well, as demonstrated through our evaluation of reach, fidelity, and acceptability, we learned that a large percentage of gardeners found getting started difficult and therefore, more personalized support to get started with periodic check-ins over the garden season would be helpful. Gardeners provided examples of support they needed to foster feelings of competency and self-efficacy including more hands-on instruction about plot preparation, plant and weed identification, soil amendments, tool usage, and watering schedules. In this trial, difficulty getting started was sometimes aggravated by delays in placement of participants in gardens, by which time weeds overwhelmed garden plots. New gardeners may feel less overwhelmed and intimidated to start gardening if given a well-maintained plot at the outset or, if such a plot is not available, providing additional in-garden support to gardeners confronted with weedy plots. Study staff were able to augment the capacity of the garden organization, occasionally providing participants with additional support and personal attention. However, to sustain garden participation for new gardeners, more organizational capacity is recommended to provide the extra time and instruction requested by new gardeners.

Through workdays, volunteer cleanups, and social events like garden potlucks, gardeners were able to connect with one another, supporting interpersonal connections. New gardeners either reported positively about these activities or reported their absence as something they missed in the garden experience. Standardizing these practices in the community garden context may be important for ensuring that gardeners feel connected to the social organization of the garden, thereby engaging and sustaining gardener participation and helping to generate connections, social cohesion, and informal social control in the garden^22^.

Environmental supports included the tools and amenities in the garden, such as access to water, tools, compost, and plant seeds and seedlings. The delivery and receipt of these amenities were implemented as planned and standardized across gardens. A subset of gardeners requested more training on the use of tools, plant identification, and the application of compost. In the Denver context, DUG is developing a range of supports to provide more attention for new gardeners, including the assignment of MCGs. In the trial, we were able to engage MCGs in the delivery of educational materials during new gardener orientation. Future work should include training about gardening across the entire season. Gardeners often asked for in-person help and mentoring in the garden, particularly when preparing their plot for the gardening season. Existing resources, including MCGs, can fulfill this need, helping gardeners start strong in the garden and giving new gardeners an initial social tie within the garden. Mentoring approaches have been implemented successfully in other gardening interventions^5,37,38^. Future evaluations could also consider the effects of structural (e.g., size of plot and type of garden arrangement) and social (e.g., leadership, mentoring, garden buddies) elements on garden participation, satisfaction, perceived success, and related psychosocial and health outcomes.

These elements of support are achievable, as demonstrated through the infrastructure and programmatic elements we evaluated in Denver, Colorado. With slight adjustments to support new gardeners, community gardening can be available to those who may feel reluctant to begin and to those who may struggle to continue. Garden organizations should be intentional about recruiting new gardeners from diverse and underserved communities, coordinating with local organizations and health clinics to raise awareness and incorporate gardens as part of a social prescribing strategy to address a range of health needs^39^, and incorporating participant engagement skills in garden leader symposia and training materials.

Maintenance of gardening is an area that requires more attention. In the CAPS trial, we followed participants over the gardening season, approximately nine months from recruitment in April to post-harvest period in January. However, we were not able to follow gardeners into subsequent gardening seasons. Gardening skills take time to develop, and the benefits of community garden participation may not be fully realized within one gardening season. Future studies are needed to follow the gardener’s trajectory and examine how intrapersonal skills, interpersonal relationships, and environmental supports evolve to promote gardening as a lifestyle activity that fosters deeper social connections, improves well-being, and benefits physical and mental health.

Staffing for the CAPS trial was consistent over the entire study, with the same investigative team and project coordinator overseeing all waves of data collection, ensuring that changes were consistent with the original study design. A manual of procedures was developed in the design phase of the study and was updated frequently to capture any changes to the study protocol. All field staff received extensive training. However, due to limited budgets, staff conducting health visits were also involved in the collection of process evaluation data, which has the possibility for measurement bias. Sekhon and others suggest measuring participant satisfaction before, during, and after the trial^31^. Given the complexity of the trial and the numerous measurement tools, we were only able to release one satisfaction survey at the end of the study, which suffered from moderate response rate (48%). Additional staff and alternative timing for survey deployment may have improved response rates for surveys and instruments that fell outside of the main data collection timepoints (baseline, harvest, and post-harvest follow-up), including the exit survey. Our measures of gardening frequency and intensity was insufficient. Diaries and check-ins were self-reported and occasionally incomplete, reducing our ability to reconstruct gardening activity and gardening “dose.” Incorporating innovative techniques such as experience sampling methods or ecological momentary assessments (EMA) could offer more robust information to support the calculation of dose by collecting real-time data^38^. Such robust data could then inform dose reconstruction related to community gardening and time spent outdoors. Finally, our final wave of data collected was interrupted by the COVID-19 pandemic. Because the impact occurred during the last follow up period, when participants were not gardening, we opted to include this time point in our analysis to provide a complete picture of the implementation of the intervention for participants. During this time, staff were in contact with participants and were able to complete data collection for the Wave 3, T3.

## Conclusion

The CAPS trial was implemented with high fidelity, supporting the validity of the trial outcomes. The trial reached the targeted mixed-income, multi-ethnic population and proved appealing among diverse groups. Core intervention elements were delivered across all sites and participants with high fidelity to the intervention protocol; fidelity could be improved with respect to uptake of gardening education and provision of social events across garden sites. This evaluation demonstrated that many new gardeners desire greater social and educational support when beginning their first gardening season. Participants found the intervention acceptable as the trial had low rates of dropouts, and participants rated most elements of the intervention favorably. We showed that community gardening is a feasible and acceptable health-promotion intervention within a diverse population. Results from this evaluation can be used to inform local and regional practices and policies supporting community gardens and nature-based social interventions more broadly. Moreover, these results are useful for municipalities, non-government organizations, and health and land agency professionals looking to advance garden programs that promote population health and engage communities in active and healthy lifestyles.

### Supplementary Information

Supplementary Information 1.

Supplementary Information 2.

Supplementary Information 3.

## Data Availability

The datasets used and/or analyzed during the current study are available from the corresponding author on reasonable request.
