# Supplementary material for: A process evaluation of a randomized-controlled trial of community gardening to improve health behaviors and reduce stress and anxiety
Source: Sci Rep. 2024 Jun 13;14:13620. doi: 10.1038/s41598-024-63889-w (PMC11176184; doi:10.1038/s41598-024-63889-w)
Supplement: Supplementary file 2 — Supplementary Information 2. [file 41598_2024_63889_MOESM2_ESM.docx]

Assessment

*Community Activation for Prevention (CAPs): A Randomized Controlled Trial of Gardening*

*Page 1 of 7*

Record ID

**Participant ID: [first_health_visit_arm_1][participant_id]**

**Community Action for Prevention: Participant Mid-Season Check-In:**

1. **To provide a mid-season check-in for all participants. For garden participants:**
2. **To understand level of involvement and participation in the community gardens**
3. **To understand barriers that prevent people from participating in their community garden**
4. **To identify issues or concerns participants may have about their participation in the community garden**

**Location of interview: At a place convenient to the participant**

- - **Via telephone or Skype (preferred)**
  - **Community garden (alternate)**

**Duration of interview:**

**Control group: approximately 5 minutes Gardening group: approximately 20-30 minutes**

**Interview process:**

1. **Attempt to make the participant feel as comfortable as possible in the current setting (if in person, either by suggesting having a seat, or walking around.)**
2. **Continue with the interview questions.**

**Suggested prompts, words to acknowledge you are listening:**

- - **Can you say more about that?**
  - **Tell me more about that?**
  - **That's interesting, what do you mean by that?**
  - **That must have been difficult.**
  - **I never heard of that, could you explain that a little bit more?**
  - **Is your experience typical?**
  - **Wow!**
  - **Uh hum...**
  - **Okay...**
  - **Really?**

Control


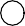

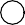


Select type of participant: Gardening

Has this interview been selected to be recorded? Yes No

(Only mark yes for selected gardening participants, for all control participants choose no.)


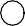

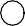


Introduction:

Hello and thank you for being part of the CAPS project! My name is and I am calling to do a quick check-in with you about the project and see how you are doing? Is now a good time?


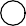
 Yes
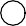
 No

Introduction:

Hello and thank you for being part of the CAPS project! My name is and I am calling to see how your garden is doing and have questions. This survey will help the study team and DUG learn about your experience with your garden and garden community. Do you have 15-20 minutes to answer these questions?


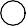
 Yes
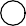
 No

**If participant is unable to answer the questions at the time of the initial phone call, ask them the following question and set up a calendar event in the CAPS google calendar. Label the participants by their ID ONLY and not by their name.**

**o When would be a better time for us to talk?**

- **If participant isn't specific with available times, ask:**
- **What's a good day to talk this week?**
- **And a good time?**

**If participant is able to answer the questions at the time of the phone call, continue with the script and survey:**

**As I go through these questions, please stop me at any time if you have any questions or if you need clarification. You do not have to answer any questions that you don't want to and you can tell me to "skip it" and I'll move on to the next question.**

We would like to audio record this interview for quality control purposes. Neither your name or any other identifying information will be associated with the audio or audio recording or the transcript. Only the research team will be able to listen to the recording.

The tapes will be transcribed by the research team and erased once the transcriptions are checked for accuracy. Transcripts of your interview may be reproduced in whole or in part for use in presentations or written products that result from this study. Neither your name or any other identifying information (such as your voice) will be used in presentations or in written products resulting from the study.

By consenting to be recorded, you are allowing the research team to audio tape you as part of the research. Your consent for this recording is effective until December 31st 2020. On or before that date, the recording will be destroyed.

Do we have your permission to begin the recording? Y/N

If at any time during the interview, you would like us to turn off the tape recorder or stop recording, that's okay, just let me know.


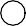
 Yes
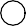
 No

How is your summer going?

Do you have any questions about the CAPS study?

(If yes, enter text of response here. If no, then skip)

Next, I will ask you questions about your overall experience at the community garden and about your interactions with other gardeners at the community garden.

OVERALL EXPERIENCE AND GARDEN PLOT:

Tell me about your experience in the community garden so far. Let's start with your garden plot.

What, if anything, did you do to prepare your soil for the growing season? Probes:

- - Did you add compost, did you turn the soil, did you weed, did you add mulch to conserve water?

What have you planted so far?

Probes:

- - Did you plant any of the seeds that DUG and the CAPs study gave you? What else have you planted? Where did you get the other seeds/ plants?

Was there anything difficult about getting your garden plot started?
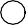
 Yes
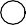
 No

If yes, please describe what was difficult.

Have you been able to pick any of the food you've grown?
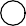
 Yes
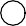
 No

If yes, what have you harvested?

Have you had adequate access to water for your plot?
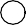
 Yes
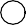
 No

If no, please explain:

Do you have any concerns about the quality of the soil in your community garden plot?

Probes

- - Toxicity
  - Nutrients
  - Physical hazards (glass, trash, etc.)

COMMUNITY GARDEN ATTENDANCE:

Thank you for sharing your experience with your garden plot and community garden. This next set of questions are about the times that you visit your garden. There is no right or wrong amount of time to visit. Everyone is different and we want to learn about the time you spend at the community garden.

When do you generally go to the garden?

If participant is struggling to give a response, ask them:

- - What days of the week do you go to the garden?
  - What time of day do you typically visit the garden?

Generally, how long do you spend at the garden each time you go?

When you are at the garden, what are some of the things that you do as part of your routine?

Have there been any challenges getting to and/or participating in your community garden?
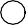
 Yes
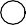
 No

If so, what are the challenges?

If they say time, you must probe further and ask:

- - "How important is it for you to garden?"
  - "Why was it of interest to join the study?"
  - "Is there anything other than time that is preventing you from gardening?"

Generally, do you feel safe when you are at the community garden?

Probes:

- - Getting to garden?
  - Being at the garden?
  - Fenced
  - Safety of roads and sidewalks
  - Personal safety

Can you describe the level of communication with the garden leader and how you have communicated with them?

Probes:

- - Have you been getting updates on the garden and events? Y/N
  - If so, how often? Daily, weekly, monthly, once in a while, not at all

Frequency:


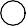
 Daily
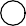
 Weekly
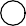
 Monthly
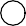
 Once in a while
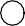
 Not at all

Type of contact:

In what ways have you interacted with the garden leader(s)?

Probes:

- - Did they help you get oriented and set up at the garden?
  - Have you worked with them at garden events?
  - Did they help you set up your plot?

Message board at the community garden Email list/ Newsletter

Individual emails Calendar of events Phone calls

Text messages In-person

Website or Facebook page other (specify in comment field)


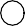

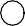


Enter comments about garden leader interaction here, including specific "other" interaction with garden leaders

How often do you see or hear from other community Daily

gardeners? weekly


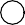

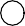

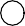

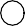

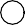


monthly

once in a while not at all


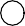

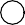


Have there been garden workdays, pot-lucks, or other Yes events at the community garden? No

Have you been able to go to events at the community Yes garden? No

If yes, how often do you go?

all of the events


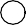

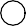

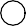


the majority of the events some of the events

And which ones have you gone to?

Over the past two weeks, can you describe the types of interactions you have had with other gardeners? Some examples of interactions could be things like saying "hello," talking about gardening, introducing each other, and asking questions.

Probes:

- - If you have questions about the garden, do you ask questions or ask for help in the community garden?
  - Have you helped anyone else in the garden with their plots or other tasks around the garden like composting or watering?

Some community gardens have garden work teams, like bee teams, composting teams, and watering teams, that help with maintaining the community garden. Are you a part of a work team or do you have a job or responsibility at your community garden?

Yes No


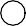

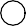


If yes, what team or responsibilities do you have?

Do you need any tools or support for your garden that you don't already have?

Yes No


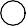

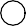


If yes, what do you need?

Is there anything else the CAPs study team can do to help?

Yes No


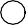

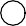


If yes, what type of help do you need?

Do you think the expectations at the garden are reasonable?

Yes No


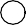

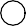


If not, how would you change things to make them more reasonable?

GARDEN CLASS FEEDBACK:

In the beginning of the gardening season, the CAPS team offered a gardening class and provided every participant with a packet of informational sheets that included information on getting your garden started.

Were you able to attend one of the gardening classes Yes


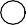

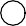


No

Do you feel it helped you think about how to get started in your garden plot?

Yes No


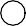

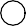


If no, what could we additional information would you have liked to have?

Were you able to look over the packet of information that the CAPS team provided?

Yes No


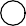

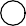


If yes, did the information provided help you learn about how to maintain your garden plot?

Yes No


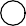

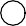


If yes, how did the information provided help you maintain your garden?

Closing the interview:

1. Thank the participant for their time and explain to them that they have been entered for a chance to win at the yearly drawing, happening in November.
2. Ask them if they have any questions for you about the study or anything in general.
3. Reminder about T2 and explain timeline and scheduling. Ask about any travel plans that we need to plan for in setting up follow up appointment.
