# Supplementary material for: A process evaluation of a randomized-controlled trial of community gardening to improve health behaviors and reduce stress and anxiety
Source: Sci Rep. 2024 Jun 13;14:13620. doi: 10.1038/s41598-024-63889-w (PMC11176184; doi:10.1038/s41598-024-63889-w)
Supplement: Supplementary file 3 — Supplementary Information 3. [file 41598_2024_63889_MOESM3_ESM.docx]

CAPS Community Gardening Study Participant Process Evaluation and Garden Continuation Survey

1. Did you participate in any form of gardening since the beginning of the study? This can include gardening at a community garden, at home, or another location (for example, at a family member or neighbor's house, or at a church).
   1. Yes, please check all that apply

i. Year 1 (2017)

| Community Garden | Home Garden | Other Location |
| --- | --- | --- |
| Flowers | Flowers | Flowers |
| Herbs | Herbs | Herbs |
| Vegetables | Vegetables | Vegetables |
| Fruits | Fruits | Fruits |
| In a garden plot or a raised bed | In a garden plot or a raised bed | In a garden plot or a raised bed |
| In a container/pot | In a container/pot | In a container/pot |

If other location, please tell us where you gardened: _______________________

ii. Year 2 (2018)

| Community Garden | Home Garden | Other Location |
| --- | --- | --- |
| Flowers | Flowers | Flowers |
| Herbs | Herbs | Herbs |
| Vegetables | Vegetables | Vegetables |
| Fruits | Fruits | Fruits |
| In a garden plot or a raised bed | In a garden plot or a raised bed | In a garden plot or a raised bed |
| In a container/pot | In a container/pot | In a container/pot |

If other location, please tell us where you gardened: _______________________

iii. Year 3 (2019)

| Community Garden | Home Garden | Other Location |
| --- | --- | --- |
| Flowers | Flowers | Flowers |
| Herbs | Herbs | Herbs |
| Vegetables | Vegetables | Vegetables |
| Fruits | Fruits | Fruits |
| In a garden plot or a raised bed | In a garden plot or a raised bed | In a garden plot or a raised bed |
| In a container/pot | In a container/pot | In a container/pot |

If other location, please tell us where you gardened: _______________________

- 1. No
     1. [Branching logic= ‘question1=2’]If you did not garden at all:
        1. Why did you decide not to garden? [Text box]
        2. *Skip to question on interest in future follow-up*

1. If you checked that you **did** garden during any of the years mentioned above, did you ever skip a year or decide to stop gardening?

Yes No

[Branching logic: if yes, the following will appear. If no, skip to question 3]

- 1. Please select all that apply.

          It required more time than I expected

          My schedule changed

          I moved

          The garden was not in a convenient location for me

          Too many pests

          I didn’t know how to manage pests

          Too many weeds

          My plants had problems that I did not know how to address

          My plants did not grow

          Weather, like heat or hail, damaged my plants and they did not recover

          I did not know how to care for my plants

          I did not know how to reach out for help with my plot

          I reached out for help with my plot but did not get a response

          I didn’t know how to prepare the food I grew

          Other; please describe: _______________________________

1. Gardening can be challenging. During any season that you gardened, did you stop gardening before the end of the season?

Yes No

[Branching logic: if yes, the following will appear. If no, skip to question 4]

- 1. If you did not garden for the whole season at any point (stopped gardening before October) we would like to know why. Please select all that apply.

          It required more time than I expected

          My schedule changed

          I moved

          The garden was not in a convenient location for me

          Too many pests

          I didn’t know how to manage pests

          Too many weeds

          My plants had problems that I did not know how to address

          My plants did not grow

          Weather, like heat or hail, damaged my plants and they did not recover

          I did not know how to care for my plants

          I did not know how to reach out for help with my plot

          I reached out for help with my plot but did not get a response

          I didn’t know how to prepare the food I grew

          Other; please describe: _______________________________

1. Please select the statement that best fits your situation for the 2020 gardening season: (*Please select all that apply*)
   - 1. I am planning to garden at a **Community Garden.**
     2. I am planning to garden at **home.**
     3. I am planning to garden at **another location**.
        1. [Branching logic] Where? ____________
     4. I am **NOT** planning to garden in the 2020 season
     5. I am unsure if I want to garden in 2020 due to COVID-19 (Coronavirus)
     6. I would like to garden in 2020, but don’t know where or need help starting out.
        1. [Branching logic=unknown if possible, in redcap] If you would like help or have questions about gardening this season (2020), would you like one of our CAPS study assistants to reach out to you?
           1. Yes, please contact me
           2. No, thank you

The following questions will ask about your experience in the **community garden**. Please answer these questions based on your personal experience.

1. Because I gardened…

|  | Strongly Disagree | Disagree | Agree | Strongly Agree |
| --- | --- | --- | --- | --- |
| I know more about gardening |  |  |  |  |
| I have more gardening skills |  |  |  |  |
| I have become more physically active |  |  |  |  |
| I ate more fruits and vegetables |  |  |  |  |
| I met new people |  |  |  |  |
| I feel happier |  |  |  |  |
| I feel socially connected to the garden community |  |  |  |  |
| I feel more connected to my neighborhood |  |  |  |  |

1. There are many things that can make a community garden successful. Please rate the following parts of the **community garden** based on your experience:

|  | Very Dissatisfied | Dissatisfied | Satisfied | Very Satisfied |
| --- | --- | --- | --- | --- |
| Seeds and seedlings provided |  |  |  |  |
| Gardening classes and workshops |  |  |  |  |
| The quality of soil in the garden plot |  |  |  |  |
| Tools available at the garden |  |  |  |  |
| Access to water |  |  |  |  |
| Lighting at the garden |  |  |  |  |
| Convenience of getting to the garden |  |  |  |  |
| Personal safety in the garden |  |  |  |  |
| Respect for plants and property in the garden |  |  |  |  |
| Opportunities to interact with other gardeners |  |  |  |  |
| Social events organized at the garden |  |  |  |  |
| Garden leadership |  |  |  |  |
| Opportunities to learn from other gardeners |  |  |  |  |
| Overall satisfaction with the garden |  |  |  |  |

1. Was there anything that kept you from being as successful at gardening as you would have liked? Please check all that apply.

          Lack of time to garden

          The garden was too far away

          Lack of gardening knowledge

          Finances

          Lack of access to water

          Lack of communication with the garden leadership

          Presence of bugs or weeds

          Weather

          Theft or vandalism within the garden

          Lack of information or guidance

          Physical ability to garden

          Other gardeners not participating in the garden

          Other; please describe ­­­­_________________________________________

          None

1. Is there anything else you would like to share with us about your experience in the garden, your experience in the study, or your future gardening plans? [text box]

____________________________________________________________________________

*Text Box/New Section:*

Would you be willing to be contacted for future studies on gardening? Additional compensation will be provided.

1. Yes, I’m interested!
2. No, thank you.

Thank you for your participation in CAPS and for completing this survey!

Sincerely,

CAPS Team
